# A qualitative study on promoting reablement among older people living at home in Norway: opportunities and constraints

**DOI:** 10.1186/s12913-022-07543-z

**Published:** 2022-02-04

**Authors:** Eliva Atieno Ambugo, Imran Dar, Mariya S. Bikova, Oddvar Førland, Trond Tjerbo

**Affiliations:** 1 Department of Health, Social and Welfare Studies, Faculty of Health and Social Sciences, University of South-Eastern Norway in Vestfold, Postboks 235, 3603 Kongsberg, Norway; 2 Department of Health Management and Health Economics, Institute of Health and Society, University of Oslo, Postboks 1089 Blindern, 0317 Oslo, Norway; 3 Northern Norway Regional Health Authority (Helse Nord RHF), Postboks 1445, 8038 Bodø, Norway; 4 Centre for Care Research Western Norway, Western Norway University of Applied Sciences in Bergen, Årstadveien 17, 5009 Bergen, Norway

**Keywords:** Older people, Reablement care, Person-centeredness, Activities of daily living, Functional limitations, Homecare services

## Abstract

**Background:**

Healthcare services that traditionally have been provided in long-term care institutions in Norway are increasingly being delivered at home to a growing population of older people with chronic conditions and functional limitations. Fostering reablement among older people is therefore important if they are to live safety at home for as long as possible. This study examines how healthcare professionals and managers (staff) in Norwegian municipalities promote reablement among community-dwelling older people.

**Methods:**

Face-to-face, semi-structured interviews lasting between 21 and 89 min were conducted between November 2018 and March 2019 with healthcare managers (*N* = 8) and professionals (*N* = 8 focus groups with 2–5 participants) in six municipalities in Norway. All interviews were audio-recorded, transcribed, and thematically coded inductively and analyzed with the aid of NVivo 12 software.

**Results:**

Overall, healthcare staff in this study used several strategies to promote reablement, including: carrying out assessments to evaluate older people’s functional status and needs (including for safe home environments), and to identify older people’s wishes and priorities with regard to reablement training. Staff designed care plans informed by the needs assessments, and worked with older people on reablement training at a suitable pace. They promoted among older people and staff (within and across care-units) the principle of ‘showing/doing with’ versus ‘doing for’ the older person so as to not enable disablement. Additionally, they supported older people in the safe and responsible use of welfare technology and equipment. Even so, staff also reported constraints to their efforts to foster reablement, such as: heavy workload, high turnover, insufficient training in reablement care, and poor collaboration across care-units.

**Conclusion:**

Older people may be supported to live safely at home by meeting them as individuals with agency, identifying and tailoring services to their needs and wishes, and encouraging their functional abilities by ‘showing/doing with’ versus ‘doing for them’ when possible. The healthcare professionals and managers in this study were positive towards reablement care. However, meeting the resource demands of reablement care is a key challenge.

## Introduction

Norway, like many other countries, has a growing elderly population who face a heightened risk of illness, disability, and dependency in carrying out basic/personal and instrumental activities of daily living—collectively referred to as ADLs henceforth [1, 2]. Increasingly, these older people are being supported to live at home for as long as possible supported by health and social care services in the community [3, 4]. One such service is ‘everyday rehabilitation’ (hverdagsrehabilitering), a Scandinavian healthcare service concept related to reablement care [5–7]. Reablement, also known as restorative care, was introduced in England and some other western countries around 2000 [8–10], and in the Scandinavian countries from 2010 [7]. Researchers ( [9] p.11) have recently endeavored to formulate an agreed-upon definition of it based on knowledge from research and practice, as follows: “Reablement is a person-centred, holistic approach that aims to enhance an individual’s physical and/or other functioning, to increase or maintain their independence in meaningful activities of daily living at their place of residence and to reduce their need for long-term services.”

Reablement care is primarily (but not exclusively) provided to older people who have the potential of benefiting from it [11]. Staff working in home-based healthcare services encompassing reablement care include physiotherapists, nurses, healthcare assistants and social workers among others [12]. Several factors have influenced growth in reablement and other homecare services in Norway and abroad, including rising healthcare costs [13], older people’s preference to live at home [13–15], and increased focus on active ageing [16–18]. The concept of reablement can be seen as a part of the broad active ageing concept adapted to a specific field within the health and social care services, namely home care services [11, 19]. It can be viewed as both a professional and political paradigmatic shift from a ‘help’ to a ‘self-help’ approach in long-term care and home care services [20]. The traditional/help approach is related to an understanding of older people as individuals with potentially increasing ageing-related care needs, whereas in the new paradigm (self-help approach), there is an expectation of people being active as long as possible throughout the life course [20]. Active ageing can also be connected to a growing trend towards living at home as long as possible and receiving individually adapted services when needed; and seen as a shift towards greater responsibility to older people themselves and to their relatives [21].

Findings on the effects of reablement care inconclusive, as evaluated via a Cochrane systematic review [11]. Even so, two Norwegian randomized controlled studies point to some beneficial effects of reablement versus usual care on older people’s activity performance and satisfaction therewith [22, 23]. Qualitative studies have also investigated pertinent topics such as establishing reablement services [24]; interdisciplinary collaboration [12, 25]; and experiences of care recipients [26, 27], family caregivers [28, 29]and healthcare personnel [30–32]. More studies on reablement care processes are however needed.

### Norwegian context

Norway is traditionally classified as a social democratic welfare state with mostly publicly funded health and social care services [33, 34]. Municipalities in Norway are responsible for primary care such as homecare services (e.g., home nursing) including reablement care [7, 34]. In 2015, the Norwegian government introduced the ‘Care plan 2020’ (Omsorg 2020) for strengthening municipal healthcare services [18]. The plan identified rehabilitation aimed at promoting mastery and independence in daily life as an important priority area (among others) within health and social care services. As part of the Care plan, municipalities received funding to address rehabilitation and other priority areas, in terms of the quality and capacity of services [18].

### The current study

This study is part of a larger research project evaluating the ‘Care plan 2020’. It examines three research questions: (1) How do healthcare staff (professionals and managers) in Norwegian municipalities work with older people living at home to promote reablement? (2) How do healthcare staff work with one another to promote reablement? (3) What factors undermine staff’s efforts to promote reablement?

The investigators of a Cochrane systematic review [11] on the effects of reablement care highlighted the need for rigorously designed randomized controlled trials (RCTs), even as they recognized the challenges of implementing such trials in community settings. They identified areas within reablement care that would benefit from further research attention, including RCTs “… to identify the critical components or processes of reablement that are most effective in promoting [and/] or maintaining … independence in older adults” ([11] p.18). The current qualitative study, while not an RCT, contributes to this call by focusing attention on care delivery strategies and processes used by healthcare staff to promote reablement among older people in Norway. In so doing, this study provides insight into potentially favourable processes (and constraining factors) in reablement care from real practice settings.

## Methods

### Design and setting

Research ethics approval and consent to participate in this study are described in section 6 under declarations. This study employed a qualitative research design. We gathered data via individual and focus group interviews (described further below) from health and social care professionals and managers in six municipalities in Norway. A qualitative design was appropriate [35, 36] given the study’s focus on understanding the strategies used by healthcare staff to promote reablement among community-dwelling older people. The municipalities included in the study were selected so as to capture some of the country’s diversity, both geographically (e.g., north, south, west, east) and in terms of rural and urban regions with varying population densities, especially of the elderly. The municipalities are kept anonymous to avoid the possibility of identifying respondents.

### Data collection procedures

#### Inclusion criteria

To be included in the study, respondents had to be healthcare professionals or managers (from the aforementioned municipalities) involved with reablement services for older people (age 65+) living at home. The study employed purposive sampling [37] given its focus on this specific group of respondents (i.e., healthcare staff working within municipal homecare services).

#### Respondent recruitment

We took the following steps to identify and recruit respondents: 1) We sought out municipalities that were taking part in one of the plans in the Care plan 2020, and that also had reablement services. We ended up with seven municipalities that represent the diversity of the country whilst also taking into consideration the available financial resources for data collection. One of the seven municipalities did not participate in data collection for this study and is therefore excluded; 2) We then contacted the leaders of health and social care services in the municipalities by email or telephone, having accessed their contact information directly from the municipal webpages. We introduced them to the project and its aims and relevance, inquired about the possibility of having their reablement care staff participate in the project and, given a positive reply, requested their support in putting us in touch with the managers of reablement services in their municipalities; 3) Next, we contacted the managers via email or telephone and introduced them to the project, invited them to participate in individual interviews, and also requested their assistance in identifying and recruiting members of their staff (reablement care managers and professionals) who could participate in interviews; 4) We then scheduled the interviews.

#### Data collection

Face-to-face, semi-structured interviews were conducted individually with managers (*N* = 8; duration: 21–63 min), and via focus-group discussions with professionals (*N* = 8 focus groups, 2–5 participants per group; duration: 41–89 min). The interviews were conducted in Norwegian by the second, third and fifth authors with support from coauthors, between November 2018 and August 2019 at municipal health and social care offices. Most of the offices were located in or adjacent to healthcare institutions (e.g., nursing homes, day centers). The interviews covered the respondents’ (healthcare staff’s) descriptions of reablement and other municipal healthcare services for older people living at home, and the experiences of these staff with developing, implementing, and making improvements to the services. We interviewed managers and professionals separately to facilitate each group’s ability to participate freely, without the two groups unduly influencing each other through their joint presence during the interviews. In particular, as pertains to the current study on reablement services, open-ended questions [38] elicited staff’s descriptions of reablement services, including their ways of working with older people, and with one another, to deliver reablement care. Appendix 1 (at the end of the manuscript) includes sample interview topics from the semi-structured guide, and a description of the larger *Care Plan 2020 evaluation project* of which this study is a part. Respondents also discussed factors that have positively or negatively influenced their ability to work with older people in ways that promote reablement.

#### Characteristics of study participants

We also gathered some demographic information about the respondents in this study. As shown in Table 1, eight of them were managers and 24 were healthcare professionals. Most managers were between 35 and 44 years old, and a large proportion of professionals were either 25–34 or 35–45 years old. The overwhelming majority of the managers and professionals were female and worked full-time with permanent employment contracts. Even so, there were some managers (12.5%) and professionals (16.7%) with temporary employment contracts; and some professionals (4.2%) surprisingly reported not knowing whether or not they were employed full time. Forty-six percent of the professionals had an employment tenure of at least 4 years, compared to 25 % of the managers. Many of the respondents also reported their professional backgrounds during the interviews. The majority of them were physiotherapists, followed by occupational therapists. There were also a few healthcare workers and (practical) nurses.

**Table 1:** Characteristics of healthcare managers and professionals affiliated with reablement services for older people living at home

*Characteristic*	Managers (*N* = 8)	Professionals (*N* = 24)
	%	%
Female (/male)	87.5	91.7
Age group		
25–34	12.5	37.5
35–44	62.5	33.3
45–54	12.5	16.7
55–64	12.5	8.3
65–74	–	4.2
Employed, permanent contract (versus temporary)	87.5	83.3
Employed, full time (versus don’t know)	100.0	95.8
Employment duration		
0–1 years	12.5	8.3
2–3	62.5	37.5
4–5	–	16.7
5+	25.0	29.2
Refused	–	8.3

### Data processing and analysis

Research ethics approval and consent to participate in this study are described in section 6 under *declarations*. In Appendix 1, we describe the larger *Care Plan 2020 evaluation project* of which this study is a part. The audio-recorded individual (managers) and focus-group (professionals) interviews were transcribed verbatim. The managers’ and professionals’ interview data were then thematically analyzed [39–41] together (described further below) with the aid of NVivo 12 software. Where needed (e.g., due to non-coinciding reports from managers and professionals; findings reported by one group and not the other), we indicated the source of the finding (i.e., manager/professional; see results section). In the analyses, we paid attention to- and integrated the manifest and latent contents of the transcripts.

#### Thematic analysis

First, we (the first and third authors) read the transcripts to become familiar with the data. Then we worked systematically with the full dataset to inductively generate and collate the first set of codes, and thereby identified interesting features/elements of the dataset. We then organized these initial codes under potential themes, and collated them within those identified themes. Next, the first author reviewed the themes to identify those that were relevant for this study’s aim of examining how healthcare staff work with older people, and with one another, to promote reablement; and the facilitating/constraining factors therein. Thereafter, the author reviewed all the codes across those relevant themes. The purpose was to identify anew (for the current study) potential themes and subthemes—under which the codes were then organized and collated. The second and fourth authors reviewed and approved the coding/analyses. This study's results are thematically organized and described in the sections that follow. All authors discussed and approved the analytic strategy.

## Results

In the sections that follow, we thematically present the findings of this study on reablement care in Norway. We use female pronouns for brevity, and to keep respondents anonymous in the direct quotes. We use the term ‘staff’ to refer to healthcare professionals and managers, and the term ‘older people’ to refer to individuals age 65+ who were the recipients of reablement services in this study. Table 2 shows the topics, themes and subthemes pertinent to this study’s aim of examining how healthcare staff promote reablement among older people living at home.

**Table 2:** Study findings

Topics	Themes	Subthemes
1. Healthcare staff’s ways of working with older people to promote reablement	1. Reablement aims and needs assessment	–
	2. Providing reablement services	1. The question ‘what is important to you’, and older people’s motivation for reablement training
		2. Reablement activities and support, older people’s home environments, and use of welfare technology/equipment
		3. ‘Showing/doing with’ versus ‘doing for’ the older person
2. Healthcare staff’s ways of working with one another to promote reablement	1. Multidisciplinary reablement care teams	–
	2. Collaboration between reablement and homecare staff	–
3. Factors that undermine healthcare staff’s efforts to promote reablement	–	–

### Topic 1: healthcare staff’s ways of working with older people to promote reablement

#### Theme 1: Reablement aims and needs assessment

In describing reablement services, staff expressed that the aim of reablement is to support older people to enhance (i.e., gain, regain, maintain, improve) their functional abilities, especially as pertains to ADLs. One professional, for example, stated that reablement has to do with the “… experience of everyday mastery [among care recipients]” (professional_1, site_1). Staff conducted needs assessments to evaluate older people’s functional status and areas of difficulty, (including) with the aid of assessment instruments (e.g., Canadian Occupational Performance Measure, Short Physical Performance Battery and others). Professionals also considered assessment instruments helpful for identifying older people’s interests and priorities in daily life. In some cases, staff assessed older people by observing them demonstrate specific activities, and considered this strategy helpful for identifying areas of difficulty and potential reasons for the challenge (e.g., the physical environment). One professional described needs assessment as follows:

*We first start with «can you show me how you would have done that [task]?” to get the older person to try and do it herself. And then the functional difficulties appear. Whether it lies with [the person’s] will or the physical surroundings, we have to in a way see how [the situation] is today to be able to know how to facilitate [prepare] so that the activity can be carried out (professional_1, site_2a).*

Staff at two sites reported on the importance of pre-emptive work: getting in touch with older people and conducting need assessments as early as possible, and where needed providing reablement services or other appropriate support. Ongoing or regular needs assessment was also important for re/identifying older people’s priorities and adjusting the care plan accordingly, and for identifying and discontinuing unnecessary services, as expressed by one manager below. Related to the latter point, managers at two sites expressed that reablement services could potentially reduce demand for municipal care services.

*We try to reach older people who apply for services for the first time …, who have never received help from the municipality … and conduct a needs assessment to find out … why [for example] the person thinks she needs a [safety] alarm (manager, site_3).*

#### Theme 2: providing reablement services

##### Subtheme 1: the question ‘what is important to you’, and older people’s motivation for reablement training

Staff described reablement services as having a point of departure in goals and activities that are important to the older person—that reflect her wishes and interests in terms of training, as one manager indicated: “… we follow the recipe ‘what is important to you?’ It is the fundamental idea in all we do actually” (manager, site_2a). During conversations with older people for example, staff asked the question ‘what is important to you?’ and other open-ended questions. Professionals expressed that spending time with older people and getting to know about their lives, their earlier functional status, and the level of functioning they wished to achieve or regain—were key processes that revealed what was important to older people, including their needs and priorities that were less apparent to other care professionals (e.g., homecare staff). Conversations with older people also contributed to activating their motivation for reablement as it enabled them to entertain the possibility of getting better (improving/regaining their functioning).

Professionals expressed that conversations with older people—which also employed motivational interviewing skills—were useful for: uncovering the need to work with older people on motivation for participating in reablement activities, or identifying when some other need (e.g., poor balance) was interfering with an older person’s motivation for reablement training and should therefore be addressed first.

*Some [older people] are so blue and so down … that they need [reablement services] to get better balance … […*] *Then comes desire when they perceive that there is improvement. (professional_1, site_7).*

The conversations were also helpful for identifying older people’s resources (e.g., functional abilities), uncovering interests that they had forgotten about or set aside due to functional limitations, and appropriately guiding an older person from less to more fitting reablement goals/activities given the person’s functional status while taking care that the older person remains interested and motivated to engage in the activity. Motivational interviewing skills were especially important when working with older people who had difficulty expressing themselves.

Staff considered person-centeredness key to the reablement way of working: e.g., focusing on what is important to the older person; tailoring training to her needs, pace, and home/local environment where they are carried out; and being attentive to her varied needs. They reported that working in this way was positive and motivating (e.g., staying engaged with reablement training) for older people, some of whom experienced that many decisions were often made for them by informal caregivers.

##### Subtheme 2: Reablement activities and support, older people’s home environments, and use of welfare technology/equipment

Following needs assessment, staff developed care plans (training activities) based on older people’s needs, interests and priorities. Employing a ‘step-by-step/stage-by-stage’ approach, staff expressed that they worked with older people on reablement training and related activities at a pace suitable for the older people, thereby accommodating older people’s sometimes changing needs. One professional described the process as follows: “… to be present with older people, and they master, and then you go a little further and challenge them a little bit, and then they dare to try in the end, and then see that “I managed it”’ (professional_1, site_5). Sometimes older people only needed someone who could be present with them during a given activity or training session to help them build their confidence and feel safe/secure in performing the activity; or to help them learn how to use a particular welfare technology or equipment. Other times, they needed some form of baseline training (e.g., to improve balance) before they could embark on reablement activities geared towards ADL functioning. Thus, via these varied efforts, staff indicated that older people took progressive steps in their training with the goal of enhancing their sense of mastery and independence in ADL functioning.

In the course of delivering reablement services, staff praised older people on their efforts during training; provided them with pertinent health information, resources and guidance; and made efforts to see their potential for reablement training and support—aimed at fostering mastery irrespective of the older person’s health status, age or perceived frailty. Staff also stated that they paid attention to older people’s home environment (e.g., assessing the need for home adjustments) and supported older people in using welfare technology and equipment when necessary and appropriate (e.g., promoting functioning/not enabling dependence). Here, one professional describes the importance of judicious use of assistive equipment in reablement work:

*… it is actually best that one does all the activities of daily life without assistive equipment for as long as it is safe. … But of course when it becomes unsafe and there is risk of falling, then one must introduce the equipment and then be good about taking it away again, because once one begins to make too much use of assistive equipment, then one begins to automatically take up those habits and carry them forward. That if one sits a little in the shower, then it is perhaps a little easier for one to sit down more than is necessary when one [is] making food (professional_2, site_7).*

##### Subtheme 3: ‘showing/doing with’ versus ‘doing for’ the older person

Staff considered ‘showing’ or ‘doing with’ as opposed to ‘doing for’ older people during training to be another key feature of the reablement way of working, and they were interested in seeing it broadly disseminated (e.g., among other healthcare staff, family caregivers, the general population). Managers acknowledged that ‘doing for’ older people was not a sustainable way of working. Instead, reablement services strive to support older people to engage in reablement activities where they identify, (re) discover, and use their existing resources/abilities to enhance their functioning. Staff described the importance of working with each other and with older people to understand the importance of supporting older people to do as much as they can for themselves in order to protect their functional abilities.

*… we have to make use of older people’s resources. It is necessary that we work in this way and that everyone take up this way of thinking: don’t do everything for her, but [allow] her to do as much as possible herself. … earlier, we did very much for older people without thinking that perhaps this person can perform this task herself … […] … for example, you made her food, you helped her get dressed, shower … And perhaps instead of thinking: can we help this person train on making food herself? Can we follow her to the shower and see what she cannot do herself, and then train on that? (manager, site_5).*

Staff also expected older people to show a willingness to actively participate in reablement activities—they considered such involvement and engagement to be key for success. Professionals at one site reported terminating reablement services for older people who were not willing to actively engage in reablement work. Still, there were some reports that homecare staff (e.g., home nurses, practical assistants) and older people did not always prioritize or were not always conscientious of the reablement way of working (i.e., staff ‘did for’ older people, who accepted ‘being done for’). Some reasons for the foregoing are described in section 3c below.

### Topic 2: healthcare staff’s ways of working with one another to promote reablement

#### Theme 1: multidisciplinary reablement care teams

Staff at two sites reported that reablement teams that are multidisciplinary, and whose members have the right attitude and a willingness to engage in the reablement way of working (e.g., showing versus doing for the older person) are important for success. One manager expressed that the multidisciplinary team she was affiliated with met regularly to discuss their work and share/exchange knowledge and experience with one another. They had a shared goal of promoting sense of mastery and independence in daily living among older people irrespective of the person’s age, such that older people could be discharged from reablement services in a timely manner and with the needed support resources (e.g., information about low-threshold and voluntary services).

*… at all times we are focused on identifying and working with the [older person’s] resources. Needs assessment. And we have all these multidisciplinary discussions. So it does something with people. Competence increases. This is what we focus on the entire time, how can the [older person]—help herself as much as possible, manage to take care of herself without help from the municipality. This is our goal (manager, site_2b).*

#### Theme 2: collaboration between reablement and homecare staff

Staff described increased collaboration between care units. They expressed that homecare teams were making progress in terms of the reablement way of working, for example, giving older people the opportunity to demonstrate their functional abilities during needs assessment and generally not doing tasks for them; and staff discussing and reflecting on the reablement way of working.

*… now we talk a lot more about making time for the older person to carry out tasks themselves … So it is quite a big change of attitude in all homecare services after one began … to talk about it [not doing tasks for the person] and focus on it … […] They [staff] were confident about it and that it is possible to work that way (professional_2, site_1).*

The reablement and homecare teams at one site worked together within an integrated model, and although there was variation between homecare teams, such collaboration overall enabled homecare staff to become more knowledgeable about the reablement way of working, understand its value, and implement it. The two teams had closer contact regarding their work with older people, which helped them engage in non-redundant, complementary activities that did not undermine one another’s work.

### Topic 3: factors that undermine healthcare staff’s efforts to promote reablement

In addition to the foregoing reports of favorable factors and processes, staff also identified challenges that have undermined the reablement way of working, including: staffing and time constraints (e.g., heavy workload among homecare staff), high staff turnover, insufficient and under-resourced training in reablement; and poorly organized/coordinated services and staff schedules that do not promote the reablement way of working, and knowledge transfer between reablement and homecare staff. Poor collaboration between these care units sometimes resulted in older people receiving mixed messages: with reablement staff supporting them to ‘do as much as they can for themselves’, and homecare staff ‘doing for them’. At another site, homecare nurses received training from reablement staff to perform pre-emptive needs assessment aimed at identifying older people’s functional challenges and intervening early. However, these strategies were not effectively implemented because staff had infrequent contact with older people; and older people were not always assisted by the same professional, making it difficult for staff to identify/monitor older people’s evolving needs and intervene accordingly.

## Discussion

Reablement care is aimed at promoting functional abilities among care recipients, especially with regard to activities of daily living [9, 11]. The healthcare professionals in this study used several strategies to promote reablement among older people living at home in Norway. they performed needs assessments to evaluate older people’s functional status and needs, identified older people’s wishes and priorities with regards to reablement services, and designed care plans based on the assessments. They also assessed the safety of older people’s home environments. Assessing clients’ needs prior to providing healthcare services is important for delivering appropriate and timely care [42–44]. Additionally, older people receiving reablement care may be at a vulnerable stage in the life course with changing health and functional status. Such that regular and ongoing needs assessment becomes central to supporting them well [42–44]. Such assessments can help professionals and older people identify the type, frequency/duration and intensity of the reablement activities to be implemented [9, 42–44]. The professionals in this study reported that conducting needs assessment was a standard procedures that helped them plan for reablement care.

Working closely with older people and identifying/asking them about what is important to them, and then taking that into consideration when planning care activities, is a key part of reablement care that aids with goal setting [44–46]. It recognizes and safeguards older people’s sense of agency, empowerment, and right to self-determination by giving them the opportunity to collaborate with healthcare professionals to make informed decisions about-, manage-, and take responsibility for their own health [47–50]. Findings in this study and others [51–53] indicate that giving older people the opportunity to participate in decisions about their care is empowering and motivating for them. Professionals in this study also used motivational interviewing techniques [54–56] to encourage older people’s engagement in reablement training. Motivational interviewing is defined as “a client-centered, directive therapeutic style to enhance readiness for change by helping clients explore and resolve ambivalence” ([55]p. 91). Through their skilled conversations with older people, professionals were able to uncover and better understand older people’s interests and priorities, their motivation for reablement, and potential barriers therein that needed to be addressed.

The healthcare professionals worked with older people on reablement activities and training at a pace suitable for the older person, and applied the principle of ‘showing/doing with’ versus ‘doing for’ [9, 41, 57, 58] the person. They discontinued services no longer needed to avoid enabling disablement among older people. Tailoring care to older people’s needs is an important element reablement care [9, 24], and should ideally exhibit a holistic understanding of- and responsiveness to older people’s changing health and social care needs [5, 47–50]. Well-tailored care is therefore key to providing appropriate services that are well aligned with older people’s functional status, abilities, and potential for improvement. Such care respects the older person’s wishes, priorities, and right to self-determination—including deciding the extent to which one is involved in reablement training [47–50].

The professionals in this study designed care plans based on their assessments of older people’s needs, and made efforts to tailor reablement activities accordingly. Reablement care has only more recently been conceptualized as “… an inclusive approach irrespective of age, capacity, diagnosis or setting” ([9]p.12), and in two Australian studies, reablement care was delivered to people with dementia [59, 60]. Norway’s municipal healthcare services are generally universal, available to all residents in need of care. Professionals in this study did not report on reablement processes for specific clients. For example, especially vulnerable older people who, in addition to needing reablement care, are also struggling with social isolation/loneliness, mental health problems, or recovery from a major illness (e.g., stroke, cancer treatment). This is an area that would benefit from further research attention.

A main goal of reablement care is to maintain/promote independence in activities of daily living, broadly defined [5, 9, 42, 45]. Older people receiving reablement care are encouraged to engage in reablement activities as much as possible, they receive appropriate support during those activities, and sometimes ‘being done for’ by professionals is the appropriate and compassionate support to receive [9, 45]. In addition, older people’s engagement in reablement activities should not be coerced, and their right to self-determination should be respected and safeguarded [9, 19, 47, 49, 50]. A central feature of reablement care practiced by professionals in this study was ‘showing/doing with’ versus ‘doing for’ older people [9, 41, 57, 58]. In this way, the professionals helped promote older people’s sense of empowerment and agency to regain/maintain (some) control over their lives and functioning [47–49]. In one Danish study [50], healthcare professionals balanced between actively encouraging and facilitating older people’s engagement in reablement, but also gave them room in other cases to not engage: “faced with older people at the last stage of life, often characterized by bodily and cognitive decline, professionals often allow older people to sit back or spend their remaining time on doing what they enjoy, which is rarely cleaning”p.2037. Such reports point to the multifaceted landscape within which reablement care is delivered, and our own findings here would have been enriched by further input from professionals describing how they work with older people who, in their opinion, could be more engaged in reablement activities but chose not to do so.

Older people are best supported when provided with care that is appropriate, timely, and well-coordinated [19, 47–49, 61]. The professionals in this study made efforts to collaborate and share knowledge within and across care units (e.g., reablement teams and homecare services). Such collaboration enabled them to learn from one another, improve their competencies in reablement care, and support older people by providing them with more consistent care and messaging regarding reablement. Other studies have also reported similar benefits of collaboration in reablement care [12, 51]. Professionals in this study also reported challenges that undermined the reablement way of working and collaboration across care units, including: heavy workloads, under-resourced/insufficient training in reablement, and poorly coordinated services. One feature that commonly underlies these challenges is finances. Reablement care, with its strong emphasis on person-centeredness, may be expected to increase user satisfaction and quality of life, although the research evidence is inconclusive [11, 44, 46, 50]. It is also less clear whether reablement reflects better use of scarce primary care resources [11, 46]. Reablement, including as described by the professionals in this study, requires solid investments in adequate and skilled professionals, good and consistent routines (e.g., ongoing needs assessments and tailoring services to users’ specific and changing needs), well-coordinated services, and user involvement and empowerment, to mention a few [9]. It can therefore put professionals in a squeeze between the expectation to deliver appropriate care on one hand, and scarce municipal resources with which to do so on the other hand.

Aspects in the provision of healthcare services described by professionals, such as time constraints, inadequate staff training, high turnover, and poorly coordinated services are related to resources/capacity. Addressing needs related to capacity would therefore remove important obstacles in providing care that promotes reablement. To this end, well thought-out ways of designing and implementing the services are key. For example, if reablement is integrated with municipal care services, the reablement team can provide staff in homecare services (e.g., home nursing, practical assistance) and other care units with education and training in the reablement way of working. Then, with mentoring and consultation from the reablement team, homecare staff can provide older people with reablement training; and staff from other units can be cognizant of and apply principles of reablement when working with older people in other capacities. Thus, over time, key principles in reablement would become general knowledge: an orientation applied more broadly, and not only by a select, specially skilled few.

Reablement represents a new way of practicing rehabilitation in a home healthcare setting; and tighter financial and organizational constraints could potentially make it difficult for leaders to prioritize and promote reablement care. One reason for this is that home healthcare is required by law for Norwegian municipalities, while reablement is not. Tighter financial and organizational constraints could also affect the boundary between helping and enabling by derailing the shift towards enabling (and not ‘doing for’) older people. While the features of reablement reported in our findings are in line with existing reablement literature [9, 11, 41, 62], there is variation in what is understood as reablement care, its components and implementation [5, 11, 45]. Findings from this study and others, and The recently developed and more unified definition of reablement [9], should help advance research and practice in the field [9, 11], including in the areas of process and outcomes evaluation. A key question for further research is whether the effects of reablement warrants diverting resources from other areas to reablement care? Does reablement care create additional increases in functioning that would not have otherwise been realized given the same resource use? Is reablement care a worthwhile investment from users’, providers’, policy makers’ and taxpayers’ perspectives? Findings from this study indicate that the strategies and care processes applied by healthcare professionals to promote reablement reflected many aspects of person-centredness. This is as it should be considering that person-centredness is central to reablement care [9, 47–49]. Person-centredness “[focuses] care on the needs of individuals. Ensuring that people’s preferences, needs and values guide [care] decisions—and providing care that is respectful of and responsive to [those needs and preferences]“ [63] section 1. A main challenge of reablement care appears to be that of capacity, and working from a person-centred framework is resource demanding: it emphasizes providing care that is: responsive to the changing health and well-being needs, wishes and preferences of people; and that is holistic—attentive to the physical, emotional/mental and social well-being of people [49]. Solving the capacity challenge in reablement care, including those reported by the professionals in this study, will require increased spending among other strategies.

While there are several promising studies done on the effects of reablement [11, 22, 23], it is uncertain whether there can be a general answer to whether or not increasing resources for use in reablement will represent a better option than other alternatives. This might be a question that has to be answered on a case-by-case basis. Even so, promoting the potential and quality of life of citizens as they move through the life course, which are important aspects of reablement and active ageing [9, 11, 16, 19], should be worthy of support.

### Methodological considerations

Our findings have limited generalizability given our sample of six municipalities. We made efforts to remedy this by conducting interviews in diverse municipalities (e.g., size, urbanicity). This study was part of a larger research project evaluating the Norwegian the ‘Care plan 2020’ that, through interviews, gathered data on a broad range of topics including reablement care. Our findings therefore lack the richness and specificity that in-depth interviews accord. Even so, the results add to the knowledge base on reablement strategies and processes from real practice settings. Municipalities in Norway generally provide primary healthcare services, including homecare services—of which reablement is a part, in a manner they consider appropriate given their local needs, priorities and conditions [29]. Therefore, due to variation in the implementation of reablement care, we caution against drawing strong conclusions from our findings. The municipalities included in this study were sampled from a list of municipalities that took part in projects for developing services for people with dementia living at home. Therefore, it is possible that the municipalities in this study have a strong focus on developing primary care services, which might have positively affected reablement services (e.g., components, delivery processes). Future studies conducted in different municipalities will help confirm whether the foregoing is the case. Reablement care has an attractive ideological basis given its emphasis on person-centeredness and the ethos of ‘showing/doing with’ versus ‘doing for’ the care recipient. The professionals in this study might have therefore been positively biased in their reports of reablement services.

Data collection for this study was constrained by the availability of research funding, and by the capacity/available time of healthcare staff to participate in the project. For example, the eight focus groups in the study had between 2 and 5 participants and lasted between 41 and 89 min. Some topics, such as the importance of needs assessment in reablement care, were discussed across the focus groups; whereas the subtopic of ‘regular re-assessment of older people’s needs’ was not consistently addressed across the groups. Time and resource constraints thus affected the depth/breadth of data collection in a way that may have hindered data saturation in some cases. We recognize this as a limitation of the study.

## Conclusion and implications for practice

A growing elderly population is increasingly being supported to live at home for as long as possible. In Norway, municipalities are responsible for providing primary care including reablement services aimed at supporting care recipients to maintain/improve their ADL functional status. This study focused on the strategies and processes used by healthcare professionals and managers to promote reablement among older people living at home in Norway. The findings indicate that professionals used several strategies, including: developing care plans based on assessments of older people’s functional status and needs, and their wishes and interest; working with older people (at a suitable pace) on reablement activities that emphasized ‘showing/doing with’ versus ‘doing for’ the older person; and collaborating within and across care units to promote the reablement way of working. Healthcare professionals and managers were overall positive towards reablement care, and considered it meaningful to employ different strategies to promote reablement among older people. A main challenge however is the resource prioritization dilemma between reablement services and traditional long-term care. The very features that make reablement care attractive (e.g., user perspective and involvement, person-centeredness and tailored care, promoting/enabling ADL functioning) are the same ones that make it resource demanding in a context where the need for services exceeds the available healthcare funding.

The level of (a) symmetry between the costs and benefits of reablement care is an area that requires more research attention, and is likely to determine the future of reablement care as a lasting component of home and community-based primary healthcare services. Even so, it may be advantageous for managers, professionals and other key stakeholders in municipal healthcare services to invest in diffusing across care units the reablement ethos of ‘showing/doing with’ versus ‘doing for’ the older person—to the extent possible and appropriate. Such an effort would require generous resource investments to support needed changes and initiatives within- and across care units (e.g., staff training and follow-up, changing work cultures among staff and care recipients, reorganizing/coordinating services, monitoring/evaluating the appropriate use of resources in implementing reablement services). Although diffusion would be demanding, the hope is that ‘showing/doing with’ versus ‘doing for’ the older person would: 1) become the default way of working with care recipients where appropriate (including with regard to the person’s functional status/capacity and motivation to engage in reablement), across care units and staff; and 2) yield gains for older people’s ADL functioning and delay the need for more intensive support/nursing home placement.

## Data Availability

The data that support the findings of this study were used under license for the current study, and so are not publicly available. Data are however available from the authors upon reasonable request, and with permission from the participants and the Norwegian Center for Research Data.

## Appendix

### The Care Plan 2020 project and interview topics pertinent to the current study

A) The Care Plan 2020 evaluation project: This study is part of a larger research project evaluating the ‘Care plan 2020’ for Norwegian municipal health and social care services. The project looks at how the municipalities are adapting to demographic changes in the society, such as the growing elderly population, and is comprised of the following components: (1) a quantitative study of municipal operations and investments in health and social care services, paying attention to changes over time in factors such as personnel man-hours, number of service users, and number of places in institutions such as rooms in nursing homes; (2) a qualitative study of municipal strategies, innovations and investments around different forms of housing/care places (e.g., independent living, assisted living, residential care such as group homes, institutional care), involvement of users therein; and the experiences of users, their families, and personnel with the foregoing; (3) a qualitative study on the organization, delivery and effects of services (including innovations and improvements therein) for older people living at home with dementia, or receiving reablement services at home.

B) Data collection and the current study: This study is part of component 3. Semi-structured interview guides addressing component 3 were developed, and then tailored to the individual and focus group interviews with managers and professionals respectively. Interviews were conducted separately with healthcare staff (managers and professionals) involved with services for older people: (i) living at home with dementia, and (ii) receiving reablement care at home. The current study is about the latter. In particular, it focuses on the strategies and processes used by healthcare staff to promote reablement, and facilitating/hindering factors therein. Interview data pertinent to the foregoing (and not services for people with dementia, for example) was thus analyzed for this study.

C) Interview topics: Below are examples of interview topics that were relevant for the current study, and which were tailored to the individual and focus group interviews with managers and professionals respectively.

*Regarding the implementation of reablement services:*

1. *What measures have been implemented?*
2. *How do you think the project (implementation of reablement services) has:*
  - *Changed the way you collaborate/communicate/share information?*
  - *Affected the person-centeredness of the service? What do you do to ensure that the service is person-centered (*e.g.*, with regard to needs assessment, care planning, involving users)?*
  - *Affected how prevention-oriented the service is? What does the service do to ensure that users can maintain independence and continue living at home (*e.g.*, with regard to early identification of needs, providing service users with information)?*
  - *Affected the safety/security of service users? What do you do to help users feel safe at home? (*e.g.*, with regard to fall prevention, assistive equipment/aids, information)?*
3. *How have different factors (examples below) influenced (facilitated/hindered) the implementation of reablement services?*
  - *Leadership and governance (*e.g.*, arrangements/mechanisms for optimally organising the continuum of care across domains/service units)*
  - *Organisational issues (*e.g.*, culture)*
  - *Collaboration (*e.g.*, within organizations, division of roles and responsibilities)*
  - *Availability of resources (*e.g.*, knowledge, staff, funding)*

*Looking back, what have been the three most important facilitating conditions? Barriers?*
